# The Web-Based Advance Care Planning Program “Explore Your Preferences for Treatment and Care”: Development, Pilot Study, and Before-and-After Evaluation

**DOI:** 10.2196/38561

**Published:** 2022-12-02

**Authors:** Doris van der Smissen, Judith A C Rietjens, Sandra van Dulmen, Ton Drenthen, F Ragnhild M D Vrijaldenhoven-Haitsma, Marijke Wulp, Agnes van der Heide, Ida J Korfage

**Affiliations:** 1 Department of Public Health Erasmus MC, University Medical Center Rotterdam Rotterdam Netherlands; 2 Nivel (Netherlands institute for health services research) Utrecht Netherlands; 3 Department of Primary and Community Care Radboud Institute for Health Sciences Radboud university medical center Nijmegen Netherlands; 4 Faculty of Caring Science, Work Life and Social Welfare University of Borås Borås Sweden; 5 Dutch College of General Practitioners Utrecht Netherlands; 6 Agora Bunnik Netherlands

**Keywords:** advance care planning, internet-based intervention, decision aids, patient education, eHealth, health communication, patient-centered care, chronic disease

## Abstract

**Background:**

Web-based advance care planning (ACP) programs may support patients in thinking about and discussing their preferences for future treatment and care. However, they are not widely available, and only a limited number of programs are evidence based.

**Objective:**

We aimed to develop and evaluate an evidence-based, interactive web-based ACP program that guides users through the process of thinking about, discussing, and recording of preferences for treatment and care.

**Methods:**

The program “Explore your preferences for treatment and care” was developed, pilot-tested on feasibility, and subsequently evaluated; engagement in ACP was assessed before program completion and 2 months after program completion using the ACP Engagement Survey (score 1-5) among 147 persons with chronic disease. Usability (score 0-100) and user satisfaction (score 1-5) were also assessed.

**Results:**

ACP engagement increased from 2.8 before program completion to 3.0 two months after program completion (*P*<.001); contemplation about ACP increased from 2.6 to 2.8 (*P*=.003), and readiness for ACP increased from 2.2 to 2.5 (*P*<.001). No changes were found for knowledge about ACP (3.0-3.2; *P*=.07) and self-efficacy for ACP (3.8-3.8; *P*=.25). The program was perceived as usable (mean 70, SD 13), attractive (mean 3.8, SD 0.7), and comprehensible (mean 4.2, SD 0.6).

**Conclusions:**

We developed an evidence-based, interactive web-based ACP program in cocreation with patients, relatives, and health care professionals. Before-and-after evaluation showed that the program can support people in taking first steps in ACP and in reflecting on preferences for treatment and care, by guiding them through the process of ACP using a stepwise approach. Participants perceived the program as usable and understandable, and they were satisfied with the program and with the amount of information. Health care professionals may use the program as a tool to start ACP discussions with their patients. The program may increase awareness of ACP.

## Introduction

### Advance Care Planning

Reflecting on future treatment and care can be relevant for everyone, but especially for older persons and people with chronic diseases [1]. Advance care planning (ACP) enables patients to define their goals and preferences for future medical treatment and care, to discuss these with relatives and health care professionals, and to record these in a document such as an advance directive [1]. ACP facilitates decision-making by patients, relatives, and health care professionals [1,2]. In case patients’ health condition worsens and they are unable to express their preferences themselves, it is important that their preferences are known by relatives and health care professionals to facilitate care in concordance with patients’ values, goals, and preferences for treatment and care [1,2].

ACP is typically conducted through face-to-face conversations between patients, their relatives, and health care professionals [2]. However, ACP conversations do not take place as often as patients, their relatives, and health care professionals would prefer [3,4]; patients expect health care professionals to initiate such conversations, whereas health care professionals are hesitant to do so and lack time and training [4,5].

### Web-Based ACP Programs

Interactive web-based programs may support patients in the first steps of ACP, for instance, in communicating their preferences and in recording these preferences [6]. Web-based programs can be accessed at any preferred time and place and therefore a larger audience can access ACP (information) [6]. Furthermore, as web-based programs can be tailored and delivered in an interactive and stepwise format, this may complement ACP processes as facilitated by health care professionals, for example, by supporting people in preparing discussions with their health care professionals, and consider their preferences for treatment and care, goals, and values [6].

Examples of web-based ACP programs that have been shown to support people in ACP include the *Prepare For Your Care* program [7] and the *Making Your Wishes Known* program [8]. A scoping review showed that web-based ACP programs have the potential to support people in ACP [6]. Furthermore, the scoping review showed that most evidence-based, interactive web-based ACP programs have been developed in the United States and only a few have been thoroughly evaluated [6]. Evaluation of the web-based ACP programs (including their feasibility, usability, and user satisfaction) is important to ensure the program is reliable and feasible to users. The results of such an evaluation can be used to improve web-based ACP programs and may be incorporated in other ACP interventions.

The aim of this study was to develop and evaluate an evidence-based, interactive web-based program for ACP in the Netherlands.

## Methods

### Developmental Process of the Web-Based ACP Program

The developmental process of the web-based ACP program was based on the main principles of the Medical Research Council guidance for developing and evaluating complex interventions, which consists of four phases: development or identification of the intervention, feasibility, evaluation, and implementation; while in each phase considering context, developing and refining program theory, engaging stakeholders, identifying key uncertainties, refining the intervention, and economic considerations [9]. From the start of the study, we aimed to include several stakeholders in all phases of the study (patients, health care professionals, and patient organizations) and for the program to be inclusive, including persons with low health literacy. First, we gained insight into the needs, preferences, and values of the stakeholders. On the basis of the needs, preferences, and values, we created a prototype of the web-based program in collaboration with the stakeholders. Next, we evaluated (feasibility and effect evaluation) and implemented the program.

The objectives of the program were to develop a web-based program that (1) informs about ACP and its possibilities and impossibilities; (2) invites patients to think about preferences and goals for future treatment and care; (3) invites patients and relatives to share preferences and goals for future treatment and care with each other and with health care professionals; (4) invites patients to record preferences and goals for treatment and care; and (5) invites patients to appoint a health care representative. Users could choose the steps of ACP they are ready to engage in and are not required to complete the entire program.

Input for the program’s content and structure came from several sources (Table 1):

1. In a scoping review, we examined the content, feasibility, and effectiveness of evidence-based, interactive web-based ACP programs [6]. We identified effective ACP elements, such as the exploration of values and goals, communication with relatives and health care professionals, and the recording of ACP [6]. Furthermore, we identified important functionalities of web-based programs such as the use of videos, the option to have the program content read aloud, and the option to print a document. Finally, the scoping review helped to select appropriate outcome measures for the evaluation of the web-based ACP program, for instance, engagement in ACP, the program’s usability, and the users’ level of satisfaction with the program [6].
2. In an interview study, we identified information needs of patients with chronic diseases and their relatives regarding web-based ACP, such as the need for information about ACP and its relevance, the need for reliable information about their disease and (arranging) care, and the need for peer support, as well as search terms for finding ACP related information [10].
3. A stakeholder group was formed to include the perspectives of different stakeholders during the development, evaluation, and implementation of the web-based ACP program. The stakeholder group included 1 patient (co-author FRMDVH), 2 relatives, and representatives of the Dutch College of General Practitioners (Nederlands Huisartsen Genootschap), the Dutch Association for Kidney Patients (Nierpatiënten Vereniging Nederland), the Dutch Association for Patients (de Nederlandse Patiëntenvereniging), and Agora (organization to promote the palliative approach). Furthermore, the stakeholder group included 1 expert in health communication of the Nivel (Netherlands institute for health services research), 1 expert in eHealth of the University of Twente, 1 representative of Vital Innovators, an organization that conducts Social Return of Investment analyses, and the researchers (DvdS, IJK, JACR, and AvdH) with expertise in shared decision-making, care at the end of life, and eHealth. During the entire 4-year project, the stakeholder group met approximately 2 to 3 times per year for 2-hour meetings. During these meetings, the progress of the project, preliminary results, and planned next steps were discussed. The stakeholders provided their feedback, which was processed by the researchers. The members of the group also assisted in the implementation of the program, for example, by disseminating the program, and participating in media interviews and in seminars about the program. The program was embedded in the general practitioners’ platform *Thuisarts.nl* [11] (English version: GPinfo.nl [12]). Representatives of Thuisarts.nl participated in the stakeholder group. Thuisarts.nl provides health-related information for patients and had 1.6 million unique visitors per month in 2016 [13]. It is visited by patients, and 90% of general practitioners reported to at least sometimes use Thuisarts.nl during consultations [13,14].

**Table 1:** Main content and characteristics of the web-based program and the studies these were based on.

Main elements of the web-based ACP^a^ program		Study findings
**Content**		
	Information about ACP, thinking about values and quality of life, communication about preferences with relatives and health care professionals, appointing a health care representative, recording of preferences in an advance directive, reviewing the advance directive.	Consensus definition of ACP (including ACP elements) [1,15] Scoping review [6] Interview study [10] Stakeholder group meetings
	References to other information pages and websites with information about disease, patient organizations, and peer support.	Interview study [10]
**Structure**		
	Interactive program; people can answer questions, watch videos, and click on additional information.	Scoping review [6] Stakeholder group meetings
	Option to save and print one’s responses to the questions in the program.	Scoping review [6] Stakeholder group meetings
	Functionalities such as hyperlinks to external websites, videos, and text-to-speech option.	Scoping review [6] Stakeholder group meetings
	Clear and simple structure, range of topics not too broad, text not too long (taking people with lower health literacy or computer skills into account).	Stakeholder group meetings
	Embedment in well-known and reliable general practitioners’ information platform (Thuisarts.nl [11]), possibilities to link to additional health information.	Stakeholder group meetings
	Inclusion of search terms as indicated by users (eg, “What is ACP?” “Recording of preferences”).	Interview study [10] Stakeholder group meetings

^a^ACP: advance care planning.

### Evaluation of the Web-Based ACP Program

#### Pilot Study

The program’s feasibility was evaluated in a pilot study. On the basis of the definition of feasibility of Bowen et al [6,16], we explored how users perceived the acceptability of the program, usability, and understandability of the text. A total of 6 patients with chronic diseases (aged 28-73 years) were included, including multiple sclerosis, cancer and kidney disease, and 3 physicians (1 male and 2 females, aged 47-66 years), 2 general practitioners and 1 surgeon with ACP experience. The three-step test interview method was used [17]. In step 1, we observed how the interviewees completed the program, while they expressed their thoughts out loud (concurrent think aloud) [17]. Step 2 was aimed at clarifying the expressions observed during step 1 [17]. In step 3, interviewees were asked about their experiences and opinions about the program [17]. The interviews were conducted at the participant’s home or the study center and lasted approximately 1 hour. The interviews were video recorded. The researcher watched the videos and made notes of important feedback, verbalizations, or actions by the interviewees.

The pilot study and evaluation study were approved by the Medical Research Ethics Committee of the Erasmus MC, University Medical Center Rotterdam on October 21, 2019 (MEC-2019-0590). Participants provided written informed consent. The data for the pilot study were collected in February 2020, and the data for the evaluation study were collected from April 2020 to June 2020.

#### Evaluation Study: Before-and-After Evaluation

##### Study Population and Study Design

Participants were recruited via an internet-based Dutch research portal [18]. In this portal, people can sign up to participate in research; they can collect points per completed survey, which they can exchange for a gift card. Inclusion criteria were as follows:

1. Having a chronic disease, defined as a disease that lasts 3 months or longer, is not (completely) curable, and which reoccurs regularly. Examples are chronic obstructive pulmonary disease, multiple sclerosis, and cancer. Participants with a psychological disorder or dementia were not invited to the study.
2. Participants aged ≥18 years were included.

We used purposive sampling by inviting comparable numbers of men and women, with diverse educational backgrounds, living across the Netherlands.

Members of the research portal were asked to (1) complete the baseline survey on ACP engagement, health literacy, and demographics; (2) complete the web-based ACP program and a survey on usability and user satisfaction; and (3) complete a survey on ACP engagement after 2 months. Reminders were sent if participants had not completed the measurement within 1 to 2 weeks. All participants had completed the baseline survey before the launch of the web-based ACP program.

##### Participant Characteristics

We assessed participants’ age, level of education, country of birth [19], and the type of chronic disease. We assessed participants’ level of health literacy by using the Dutch version of the Set of Brief Screening Questions on a 5-point Likert scale (1=not at all or never; 5=completely or always) [20,21]. We also asked the time it took to complete the web-based ACP program and whether they completed the program together with someone else.

##### ACP Engagement

ACP has evolved from focusing on completing advance directives to an ongoing behavior change process of considering, discussing, and recording goals, values, and preferences for treatment and care [1,15]. The goal of the web-based ACP program is to make users aware of ACP and to provide guidance in the first steps of ACP, such as thinking about preferences and how to discuss and record these preferences. We hypothesized that the program will influence attitudes toward initiating ACP and involvement in ACP. To assess the participants’ behavior change and involvement in ACP, we considered the ACP Engagement Survey to be a suitable instrument as it is about the entire behavior change process of ACP, considering that ACP is an ongoing process [22-24]. As the web-based ACP program is also aimed at informing users about ACP, it could be useful for people who are not yet familiar with ACP, including people who may not be ready for ACP. Research has shown that people need to feel some readiness to start engaging in ACP; however, the ACP process itself can have a positive influence on people’s readiness [25]. The participants in the study were recruited via a web-based research portal so they may not have had any prior knowledge of ACP; therefore, we expected a change in ACP engagement comparing baseline with a measurement 2 months after completion of the program.

Participants completed the validated Dutch ACP Engagement Survey (34 items) [22-24] before and 2 months after program completion. This survey focused on four ACP domains: (1) surrogate decision makers; (2) values and quality of life; (3) flexibility in surrogate decision-making; and (4) asking doctors questions [24]. ACP behavior change is measured by four subscales, namely knowledge about ACP (2 questions), contemplation about ACP (3 questions), self-efficacy for ACP (12 questions), and readiness for ACP (17 questions) [22-24]. The response options of knowledge, contemplation, and self-efficacy have a 3-point scale in the Dutch survey version, coded as 1=1, 2=3, and 3=5 [22]. The readiness subscale has a 5-point scale, ranging from 1=I have never thought about it to 5=I have already done it; the fifth answer option can be used to analyze specific ACP behaviors [22-24]. The total ACP engagement score is the mean score of all questions in the survey.

##### Usability and User Satisfaction of the Web-Based ACP Program

We assessed the program’s usability with the System Usability Scale (SUS; 5-point scale: 1=completely disagree to 5=completely agree) [26,27]. A total SUS score was computed using the scoring formula (score of 0-100) [26,27]. We assessed users’ satisfaction with the attractiveness and clarity of the program (4 questions), its comprehensibility (3 questions), and emotional support (2 questions) [28,29]. We also asked (1) whether participants would recommend the program to others (1=completely disagree; 5=completely agree); (2) how satisfied they were with the program (1=not at all satisfied; 10=very satisfied) with the possibility to add an explanation; and (3) their view about the amount of information the program provided (1=too little, 5=exactly enough, and 10=too much) with the possibility to add an explanation.

##### Statistical Analysis

We statistically analyzed participants’ responses on the ACP Engagement Survey using the software IBM SPSS Statistics. As the data were not entirely normally distributed, we conducted nonparametric testing with Wilcoxon Signed Rank statistical tests to compare participants’ responses on the ACP Engagement Survey before and 2 months after program completion. To see whether the readiness items indicated a behavior change since baseline, for instance, whether participants moved from the precontemplation behavior change stage to contemplation, action, or maintenance (from 1 to 2 points on the Likert scale at baseline to 3, 4, or 5 points after 2 months), we conducted McNemar tests [23]. To assess whether the change in scores was clinically meaningful, we applied the effect sizes as determined in the validation study of the original ACP Engagement Survey of Shi et al [30]. According to Shi et al [30], mean change scores of approximately 0.2 to 0.3 points are considered to indicate small effect sizes (0.20-0.49), 0.4 to 0.5 points are considered to indicate moderate effect sizes (0.50-0.79), and changes of ≥0.6 points are considered to indicate large effect sizes (≥0.80). To assess the association of level of education with ACP engagement, we performed a subgroup analysis by 2 mixed ANOVAs with a post hoc Bonferroni test. To check for selection bias, we compared age, level of education, and outcomes on ACP engagement of participants who completed all measurements with those who only completed the baseline. A power calculation indicated that we needed a sample size of 70 participants.

### Ethics Approval and Patient Consent

This study was approved by the Medical Research Ethics Committee of the Erasmus MC, University Medical Center Rotterdam on October 21, 2019 (MEC-2019-0590). All methods were performed in accordance with the relevant guidelines and regulations (Declaration of Helsinki). The study conforms with the International Committee of Medical Journal Editors (ICMJE) recommendations for the conduct, reporting, editing, and publication and for the protection of research participants. Participants were recruited via an internet-based Dutch research portal [18] and provided written informed consent. The authors confirm that all patient or personal identifiers have been removed or disguised so the persons described are not identifiable and cannot be identified through the details of the story.

## Results

### The Web-Based ACP Program

The web-based ACP program *Explore your preferences for treatment and care* [31] consists of three steps, guiding users through the following processes:

1. Thinking about preferences for future medical treatment and care
2. Discussing preferences for treatment and care with relatives and health care professionals and appointing a personal representative
3. Recording preferences for treatment and care; instructions are provided on how to record preferences in an advance directive and to review preferences (it is not possible to create an advance directive in the program itself)

Users can choose the steps of ACP they are ready for to engage in and are not required to complete the entire program. The program contains videos, questions, and links to information on health and disease. The user can print or save a document in PDF with the responses to the questions in the program. Textbox 1 shows the content of the program; Multimedia Appendix 1 presents screenshots of the program.

**Content and functionalities of the web-based advance care planning (ACP) program “Explore your preferences for treatment and care” [31].**

**Content of the program**

**Main topic (every bullet point is described on a separate page):**

**Home page**

- About the program
- About ACP
- For whom and when
- Useful websites
- Disclaimer

**Step 1: Thinking about your treatment and care preferences**

- Information about what is important in life and thinking about preferences (with video)
- Question: what is important to you?
- Question: what does this mean for your treatment and care preferences?
- Question: which care would you like to receive or not?
- Your preferences when being severely ill and when you will not recover anymore
- Question: what have you learned from previous experiences?
  - Statements:
    - I want to live as long as possible, even when my quality of life is not good.
    - I want to try various treatments, but I want to stop when my quality of life is no longer good.
    - I want to live as comfortable and free from pain as possible, even if this would mean my life would be shorter.

**Step 2: Discussing your treatment and care preferences**

- The health care representative (with video)
- Question: have you already thought about a health care representative?
- Question: who would you choose as your health care representative?
- The role of your health care representative
- Question: are there additional things you would want your health care representative to address?
- Discussing your preferences with your health care representative
- Question: what do you need to start the conversation with your health care representative?
- Discussing your preferences with your doctor
- Question: what do you want to discuss with your doctor?

**Step 3: Recording your treatment and care preferences**

- How to make an advance directive (with video)
- Question: have you already recorded your preferences in an advance directive?
- Question: what topics would you want to record in an advance directive?
- Discussing your advance directive and sharing it
- Question: when would you review your advance directive?

**End of program**

- Your answers as given while completing this program (user sees answers and can save these in PDF or print these)

**Functionalities of the program**

- Users can generate a document with the questions and their answers, this document can be printed and saved in PDF
- Users can navigate in the program: they can skip steps or can go back and forward in the program
- Users can track their progress in the program
- Users can answer open and closed questions, and statements
- The program is interactive: users can answer questions, click on links for additional information
- Users can watch videos with patient experiences with ACP
- The program refers to useful information about disease, treatment and care, information for relatives, patient associations, and peer support, partly within the website “Thuisarts.nl.”
- Audio can be used, text-to-speech option: text can be read out loud
- The program can be accessed by phone, computer, and tablet
- Clear and understandable language
- Clear structure and layout

### Evaluation of the Web-Based ACP Program

#### Pilot Study

In the pilot study, patients mentioned that the program made them think about their treatment and care preferences and they understood the questions in the program well. The participating physicians thought the program would be valuable for patients to support them in ACP. All interviewees were able to complete the program without problems. Some minor suggestions were given to the web design team. For example, sometimes interviewees clicked on a hyperlink to an external website without noticing they left the program website. Subsequently, the web design team inserted a notification, and they also applied small language improvements based on the interviewees’ feedback. All participants thought the program was interesting. Some interviewees mentioned that they would recommend the program to others.

#### Evaluation Study: Before-and-After Evaluation

##### Participant Characteristics

**Figure 1:**
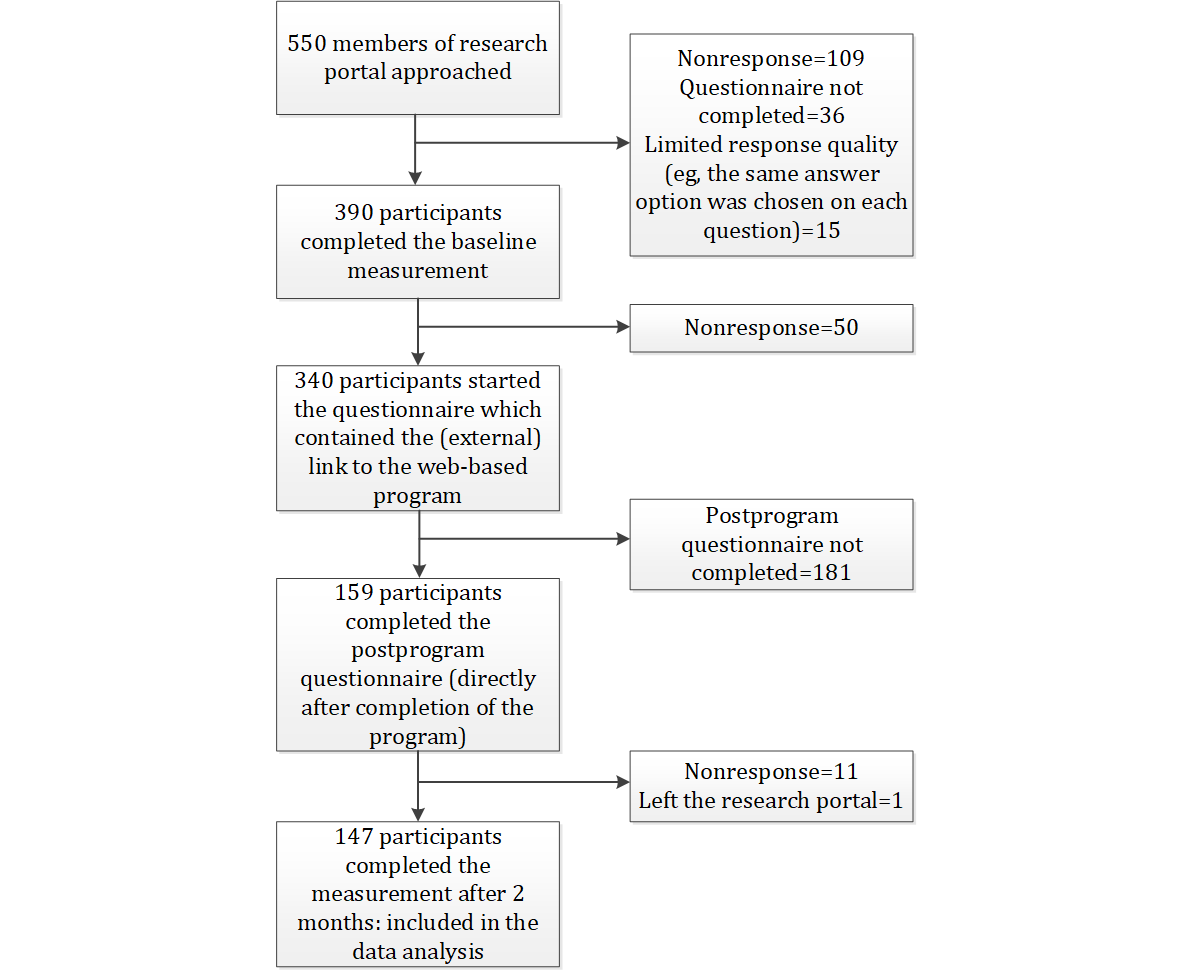
A flowchart of participants’ responses.

The baseline questionnaire was sent to 550 members of the research portal with chronic disease. The baseline measurement was completed by 70.9% (390/550) of the participants, the second measurement (program completion and additional questionnaire) was completed by 40.8% (159/390) of the participants, and the measurement after 2 months was completed by 92.5% (147/159) of the participants (Figure 1).

Participants who completed all questionnaires (n=147) were included in this study. They were 60.5 years of age on average (SD 10.7, range 26-82 years), and of the 147 participants, 82 (55.8%) were male and 65 (44.2%) were female; this was largely representative for the general Dutch population of people aged ≥18 years (with 49% males and 51% females in 2019) [32]. Of the 147 participants, 143 (97.3%) were born in the Netherlands. Levels of education differed: low, 36 (24.5%) participants; medium, 61 (41.5%) participants; and high, 50 (34%) participants; this was largely representative for the general Dutch population of people aged ≥18 years (with 28% low, 43% middle, and 29% highly educated in 2019) [32]. Levels of health literacy were high on average (mean 4.5, SD 0.5; scale 1-5). Participants completed the program within 26.1 minutes on average (SD 22.2 minutes). Two participants completed the program together with a family member or partner.

##### ACP Engagement

The total ACP engagement score increased from 2.8 before program completion to 3.0 after 2 months (*P*<.001). Contemplation about ACP increased from 2.6 to 2.8 (*P*=.003). Readiness increased from 2.2 to 2.5 (*P*<.001). No changes were found for knowledge about ACP (3.0-3.2; *P*=.07) and for self-efficacy for ACP (3.8-3.8; *P*=.25). Comparing baseline and the measurement after 2 months, we found significant increases for three of four domains: (1) surrogate decision makers increased from 2.8 to 3.0 (*P*<.001); (2) what matters most in life: health situations increased from 3.0 to 3.2 (*P*=.003) and care at the end of life increased from 2.9 to 3.0 (*P*=.02); and (3) flexibility in medical decision-making increased from 2.6 to 2.9 (*P*<.001). We found no significant differences for domain 4, ask your doctors questions (3.1-3.2; *P*=.20). Table 2 presents the results. According to the validation study of the original ACP Engagement Survey, the changes in scores indicate clinically meaningful changes [30].

The McNemar test showed no significant difference in participants’ stages of behavior change after 2 months compared with baseline (*P*=.11), except for the domain “flexibility in medical decision-making”; participants moved from precontemplation to higher stages of behavior change (contemplation, action, or maintenance); this difference was significant (*P*=.04).

The mixed ANOVAs with a post hoc Bonferroni test indicated no significant differences on level of education (low, middle, and high) for the subscales (knowledge: *P*=.06; contemplation: *P*=.51; self-efficacy: *P*=.90; readiness: *P*=.19; and total ACP engagement score: *P*=.56) and neither for the domains (surrogate decision makers: *P*=.49; what matters most in life: health situations: *P*=.41; and care at the end of life: *P*=.55; flexibility in medical decision-making: *P*=.39; and ask your doctors questions: *P*=.82).

The incomplete responses or dropout in the measurement in which the program needed to be completed was 53.2% (181/340) of participants; they started the questionnaire, which contained the (external) link to the web-based ACP program, but did not complete the postprogram questionnaire. As user data are not recorded in the program, we were not able to see whether these participants completed the program. When comparing the 147 participants who completed all measurements with the 390 participants who completed only the baseline, we found no significant differences among the groups for age (*P=*.19), education level (*P*=.29), and levels of ACP engagement (the subscales: knowledge, *P*=.92; contemplation, *P*=.34; self-efficacy, *P*=.40; readiness, *P*=.61; and the total ACP engagement score, *P*=.81) and the domains: surrogate decision makers, *P*=.98; what matters most in life: health situations, *P*=.74; and care at the end of life, *P*=.99; flexibility in medical decision-making, *P*=.43; and ask your doctors questions, *P*=.33), suggesting there was no selection bias owing to the dropout.

**Table 2:** Results of the ACP^a^ Engagement Survey (34 items) per subscale and domain (N=147).

Item				Before the ACP program, mean (SD)		2 months after the ACP program, mean (SD)		*P* value
**Subscale^b^**								
	Knowledge about ACP			3.0 (1.4)		3.2 (1.2)		.07
	Contemplation about ACP			2.6 (1.1)		2.8 (1.2)		.003
	Self-efficacy for ACP			3.8 (0.8)		3.8 (0.9)		.25
	Readiness for ACP			2.2 (1.0)		2.5 (1.1)		<.001
**Domain^b^**								
	Surrogate decision makers			2.8 (1.0)		3.0 (1.0)		<.001
	**What matters most in life**							
		Health situations	3.0 (0.9)		3.2 (0.9)		.003	
		Medical care at the end of life	2.9 (0.9)		3.0 (1.0)		.02	
	Flexibility in medical decision-making			2.6 (1.0)		2.9 (1.0)		<.001
	Asking your doctors questions			3.1 (1.0)		3.2 (1.0)		.20
Total of all questions in the ACP Engagement Survey				2.8 (0.8)		3.0 (0.9)		<.001

^a^ACP: advance care planning.

^b^The ACP Engagement Survey evaluates 4 behavior change constructs (the subscales) within 4 the ACP domains—scale 1 to 5.

##### Usability of the Web-Based ACP Program

Of the 147 participants, 50 (34%) participants indicated they would use the program frequently, 25 (17%) participants would not and 72 (49%) participants were neutral. Of the 147 participants, 115 (78.2%) thought the program was easy to use and 26 (17.7%) participants were neutral. In total, of the 147 participants, 96 (65.3%) participants thought the functions were well-integrated and 115 (78.2%) participants felt they did not need to learn a lot before they could use the program. The mean total SUS score was 70 (SD 13; score 0-100). Table 3 presents the results.

**Table 3:** Usability of the web-based ACP^a^ program according to the participants (N=147).

Usability	Participants, n (%)		
	Disagree^b^	Neutral^c^	Agree^d^
I think I would use this web-based program frequently.	25 (17)	72 (49)	50 (34)
I found the web-based program unnecessarily complex.	104 (70.7)	31 (21.1)	12 (8.2)
I thought the web-based program was easy to use.	6 (4.1)	26 (17.7)	115 (78.2)
I think I would need tech support to be able to use this web-based program.	123 (83.7)	13 (8.8)	11 (7.5)
I found the various functions in this web-based program were well-integrated.	6 (4.1)	45 (30.6)	96 (65.3)
I thought there was too much inconsistency in this web-based program.	116 (78.9)	25 (17)	6 (4.1)
I would imagine that most people would learn to use this web-based program very quickly.	11 (7.5)	41 (27.9)	95 (64.6)
I found the web-based program very cumbersome to use.	109 (74.1)	21 (14.3)	17 (11.6)
I felt very confident using the web-based program.	13 (8.8)	61 (41.5)	73 (49.7)
I need to learn a lot about this web-based program before I could effectively use it.	115 (78.2)	21 (14.3)	11 (7.5)

^a^ACP: advance care planning.

^b^Number of participants who scored 1 to 2 on the Likert scale.

^c^Number of participants who scored 3 on the Likert scale.

^d^Number of participants who scored 4 to 5 on the Likert scale.

##### Satisfaction With the Web-Based ACP Program

On average, participants rated the attractiveness of the program as 3.8 (SD 0.7; scale1-5), its comprehensibility as 4.2 (SD 0.6; scale 1-5), and the emotional support it provided as 3.4 (SD 0.8; scale 1-5). Of the 147 participants, 96 (65.3%) would recommend the program to others (Table 4). Participants rated their satisfaction with the program with 7.6 on average (SD 1.6; scale 1-10). Of 147 participants, a total of 80 (54.4%) participants added an explanation, of which 70 (88%) were positive, mentioning that the program was clear, easy to use, and important and that it made them think about preferences for treatment and care. Several mentioned that they would like to start with ACP. A few participants mentioned that it was confronting to complete the program or that they already arranged ACP.

Participants thought the amount of information in the program was enough (mean 5.6, SD 1.2; 1=too little, 5=exactly enough, and 10=too much). Of 147 participants total of 54 (36.7%) participants added an explanation, of which 47 (87%) were positive, mentioning the content of the program was enough and the information was clear; 6 (11.1%) participants found the information quite a lot to complete at once.

**Table 4:** Satisfaction with the web-based ACP^a^ program according to the participants (N=147).

User satisfaction			Participants, n (%)				
			Disagree^b^		Neutral^c^		Agree^d^
**Satisfaction with attractiveness (mean 3.8, SD 0.7)**							
	The web-based program is pleasant.	15 (10.2)		34 (23.1)		98 (66.7)	
	The web-based program is clear.	8 (5.4)		21 (14.3)		118 (80.3)	
	The web-based program is well-developed.	7 (4.8)		27 (18.4)		113 (76.9)	
	The web-based program is attractive.	10 (6.8)		50 (34)		87 (59.2)	
**Satisfaction with comprehensibility (mean 4.2, SD 0.6)**							
	The web-based program is understandable.	3 (2)		13 (8.8)		131 (89.1)	
	The texts in the web-based program are understandable.	2 (1.4)		11 (7.5)		134 (91.2)	
	The web-based program is easy to read.	2 (1.4)		13 (8.8)		132 (89.8)	
**Satisfaction with emotional support (mean 3.4, SD 0.8)**							
	The web-based program gives me self-confidence.	13 (8.8)		61 (41.5)		73 (49.7)	
	The web-based program gives me ease of mind.	20 (13.6)		64 (43.5)		63 (42.9)	
I would recommend the web-based program to others.		9 (6.1)		42 (28.6)		96 (65.3)	

^a^ACP: advance care planning.

^b^Number of participants who scored 1 to 2 on the Likert scale.

^c^Number of participants who scored 3 on the Likert scale.

^d^Number of participants who scored 4 to 5 on the Likert scale.

## Discussion

### Principal Findings

The web-based ACP program *Explore your preferences for treatment and care* [31] was considered usable and understandable. The program supported participants to engage in ACP and in thinking about their treatment and care preferences and to feel ready for ACP. The program supported participants to engage in several ACP domains, such as appointment of a health care representative and to think about what matters most in life. Participants were satisfied with the program and with the amount of information. The program gave almost half of the participants ease of mind, 65.3% (96/147) participants would recommend it to others.

### Strengths and Limitations

The program was evidence-based and developed in cocreation with patients, relatives, and health care professionals; their input ensured that it would meet the needs of its potential users. We had a varied sample of participants with chronic diseases with different ages and levels of education.

As user data are not recorded in the program, we were not able to see whether participants completed the program and were unable to analyze their responses. Numbers of incomplete responses or dropout were quite high in the measurement immediately following the completion of the ACP program. Filling in the measurement required participants to return to the questionnaire after completing the program on a separate website or web page. It may be the case that this was not clear to participants or, alternatively, that they thought the program was too long or too difficult. However, the response rates in the measurement 2 months after completion of the program were sufficient and rather high according to the research portal: 92.5% (147/159); and we found no significant differences in participant characteristics in our baseline measurement that suggested we had no selection bias owing to the dropout.

### Comparison With Prior Work

Most evidence-based, web-based ACP programs have been developed in the United States and only a few have been thoroughly evaluated [6]. We developed an evidence-based, interactive web-based ACP program in cocreation with patients, relatives, and health care professionals and sustainably embedded it in the frequently used and trusted general practitioners’ platform *Thuisarts.nl*. We used the ACP Engagement Survey to evaluate the program’s effects and found that it could support patients in ACP engagement. This confirmed the findings considering the web-based ACP program *Prepare For Your Care* from the United States [33-35]. We found changes in scores for contemplation about ACP, readiness for ACP, what matters most in life, surrogate decision makers, flexibility in medical decision-making, and total ACP engagement scores, which, according to the validation study of the original ACP Engagement Survey, indicated clinically meaningful changes [30].

The availability of the program on the web may improve access to ACP information at any preferred time and place; this can be important as ACP is considered a process over time. The web-based ACP program may be an addition to the traditional ACP process as facilitated by health care professionals, as it includes information, questions to be answered, and videos. We believe web-based programs should not replace discussions with relatives or health care professionals, but the program may support patients in preparing for ACP discussions [6]. Health care professionals may use the program as a tool to start ACP discussions with their patients. The program can support blended care by a combination of face-to-face conversations and the web-based ACP information; this fits within current developments of self-management and eHealth [6,36,37].

The program was launched in April 2020, and it has been frequently used (>78,000 visits by June 1, 2022).

### Recommendations for Future Research

As most participants are born in the Netherlands, we recommend to evaluate the program in persons with other countries of birth as well. In addition, since the participants were members of an internet-based research portal, their level of computer skills may be above the average skill of the Dutch population. As readiness for ACP can differ across patients [25], we recommend to examine how web-based ACP programs affect ACP discussions between patients and health care professionals.

### Conclusions

We developed an evidence-based, web-based ACP program *Explore your preferences for treatment and care* in cocreation with patients, relatives, and health care professionals. The before-and-after evaluation showed that the program can support people in taking first steps in ACP and in reflecting on preferences for treatment and care, by guiding them through the process of ACP using a stepwise approach. Participants perceived the program as usable and understandable, and they were satisfied with the program and the amount of information. Health care professionals may use the program as a tool to start ACP discussions with their patients. The program may increase awareness of ACP.

## Appendix

Multimedia Appendix 1 Example screenshots of the web-based advance care planning program "Explore your preferences for treatment and care".

## Abbreviations

ACP: advance care planning
SUS: System Usability Scale

## Data Availability

Data will be available for verification for at least 15 years. All research data will be retained and stored in a study database at the central network server of the Erasmus MC, University Medical Center Rotterdam, and can only be accessed by those specifically granted access to the database involved.
