# Supplementary material for: The Web-Based Advance Care Planning Program “Explore Your Preferences for Treatment and Care”: Development, Pilot Study, and Before-and-After Evaluation
Source: J Med Internet Res. 2022 Dec 2;24(12):e38561. doi: 10.2196/38561 (PMC9758635; doi:10.2196/38561)

Multimedia Appendix 1. Example screenshots of the web-based advance care planning program “Explore your preferences for treatment and care”. Link: https://www.thuisarts.nl/keuzehulp/verken-uw-wensen-voor-zorg-en-behandeling

Home page:

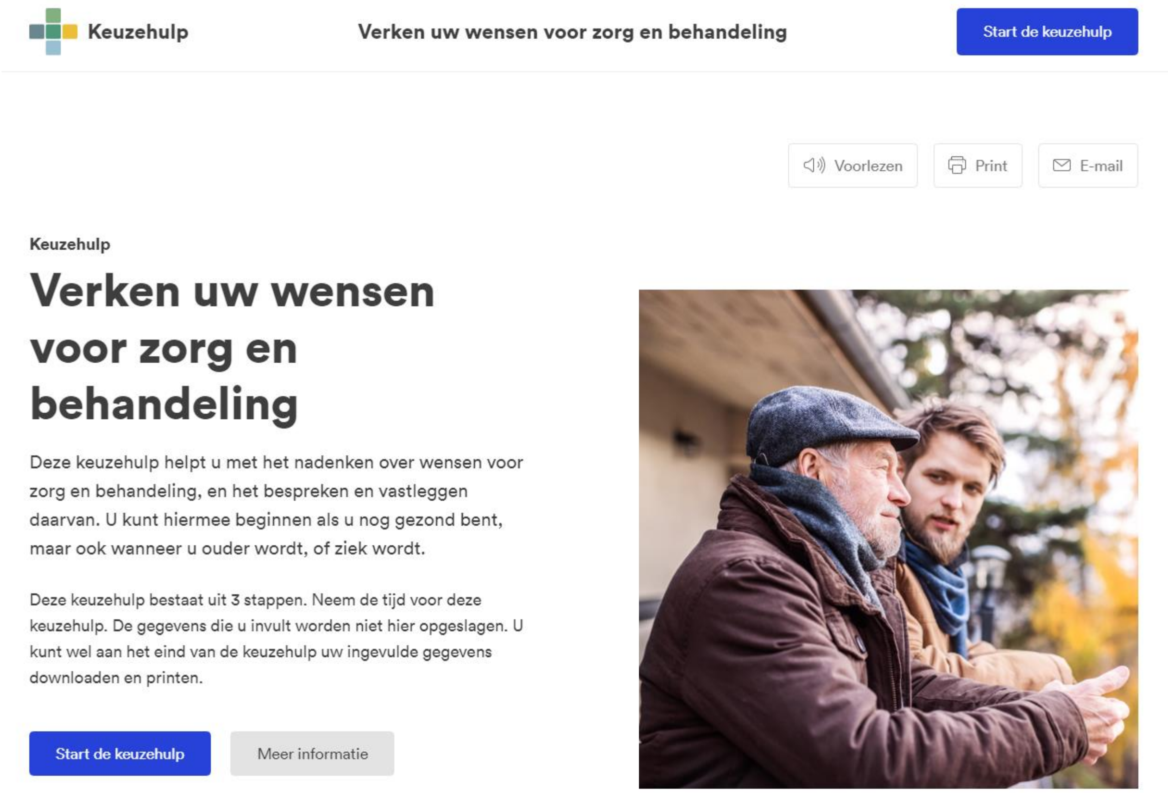

Step 1: Thinking about treatment and care preferences.

Information about what is important in life and thinking about preferences (+ video).

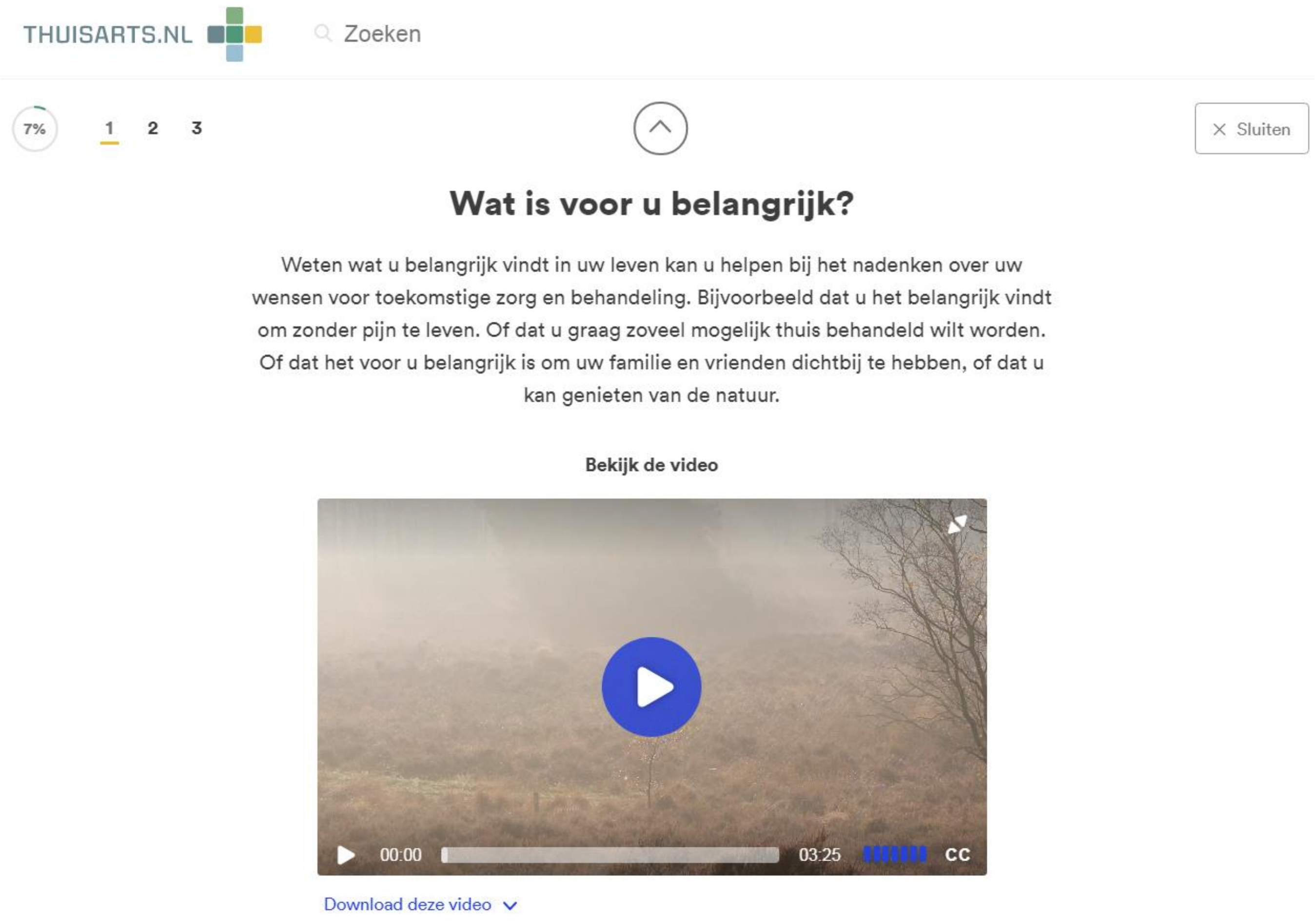
THUISARTS.NL

Zoeken

7%

1 2 3

Sluiten

Wat is voor u belangrijk?

Weten wat u belangrijk vindt in uw leven kan u helpen bij het nadenken over uw wensen voor toekomstige zorg en behandeling. Bijvoorbeeld dat u het belangrijk vindt om zonder pijn te leven. Of dat u graag zoveel mogelijk thuis behandeld wilt worden. Of dat het voor u belangrijk is om uw familie en vrienden dichtbij te hebben, of dat u kan genieten van de natuur.

Bekijk de video

00:00 03:25 CC

Download deze video

Step 1: Thinking about treatment and care preferences.

Question: Which care would you like to receive or not?

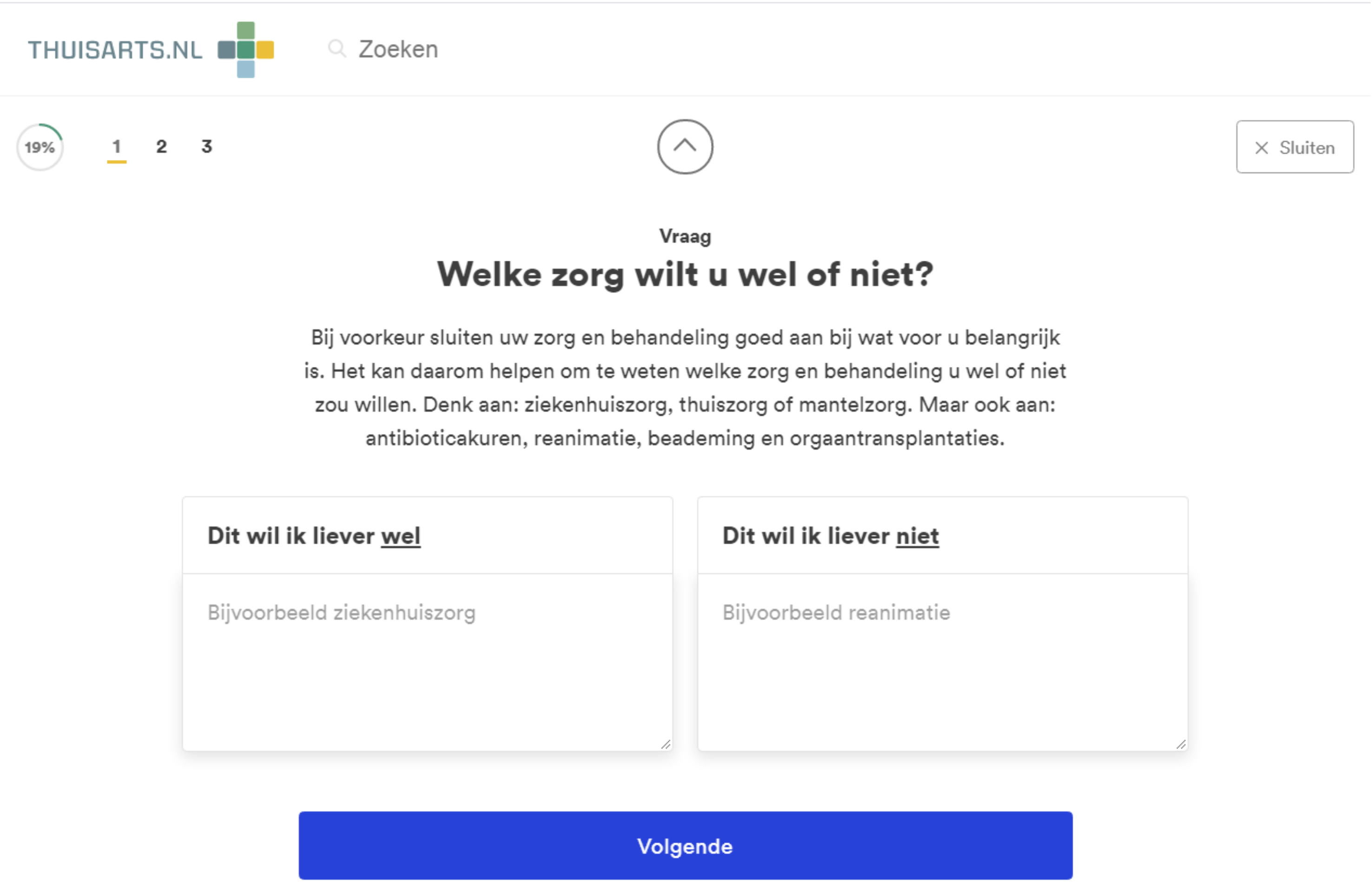

THUISARTS.NL

Zoeken

19%   1   2   3

Sluiten

Vraag

Welke zorg wilt u wel of niet?

Bij voorkeur sluiten uw zorg en behandeling goed aan bij wat voor u belangrijk is. Het kan daarom helpen om te weten welke zorg en behandeling u wel of niet zou willen. Denk aan: ziekenhuiszorg, thuiszorg of mantelzorg. Maar ook aan: antibioticakuren, reanimatie, beademing en orgaantransplantaties.

Dit wil ik liever wel	Dit wil ik liever niet
Bijvoorbeeld ziekenhuiszorg	Bijvoorbeeld reanimatie

Volgende

Step 2: Discussing your treatment and care preferences.

The healthcare representative (+ video).

THUISARTS.NL Zoeken

44% 1 2 3 Sluiten

De zorgvertegenwoordiger

Een zorgvertegenwoordiger kan uw wensen en voorkeuren namens u vertellen wanneer u dat zelf niet meer kunt, bijvoorbeeld uw partner, familielid of vriend. Kies iemand van 18 jaar of ouder, en vraag of hij of zij deze rol op zich wil en kan nemen. Kies iemand die uw wensen duidelijk kan maken, ook wanneer hij of zij het niet daarmee eens is, en ook in moeilijke situaties. Bespreek ook wie eventueel uw zorgvertegenwoordiger kan vervangen. Vertel uw naasten en arts wie uw zorgvertegenwoordiger is. Hieronder kunt u een video bekijken over het aanwijzen van een zorgvertegenwoordiger.

Ik wil meer uitleg

Bekijk de video

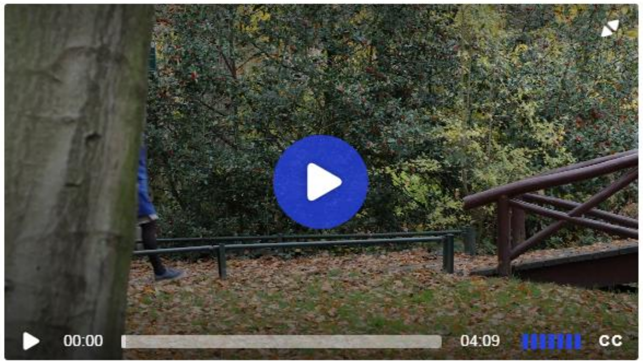

Step 2: Discussing your treatment and care preferences.

Question: What do you want to discuss with your doctor?

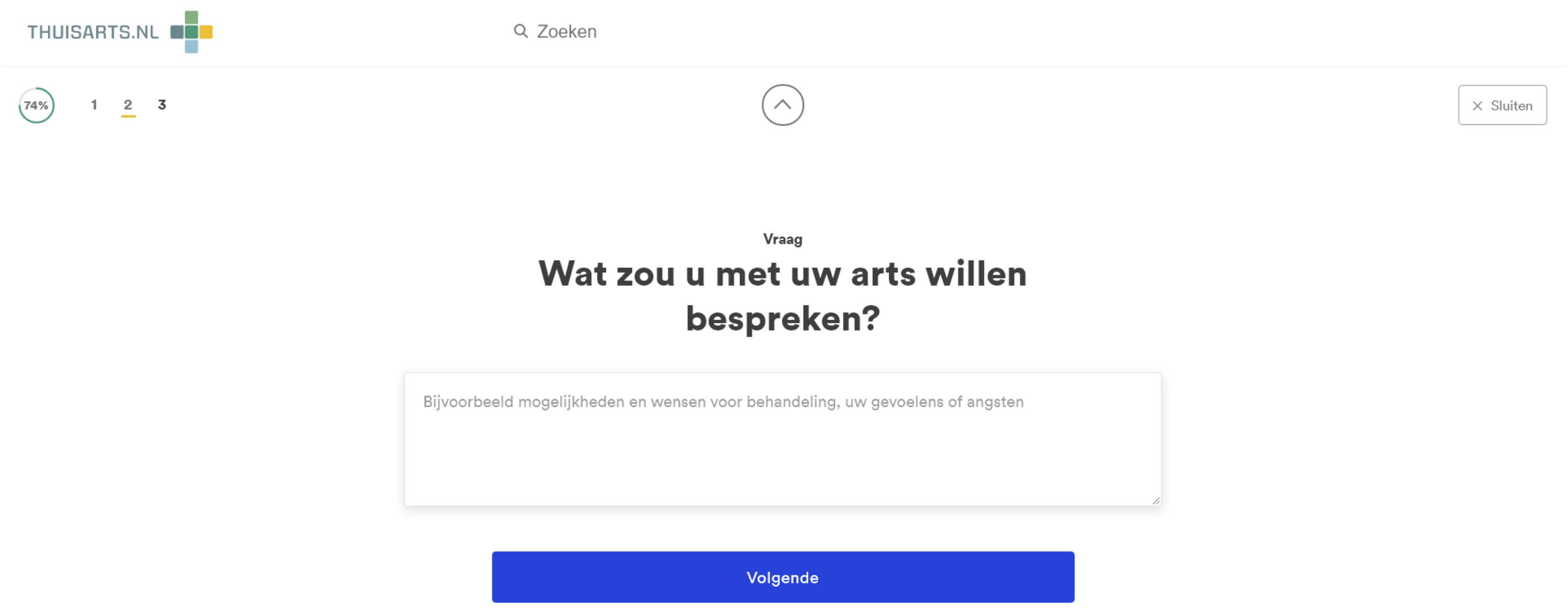

Step 3: Recording your treatment and care preferences.

How to make an advance directive (+ video).

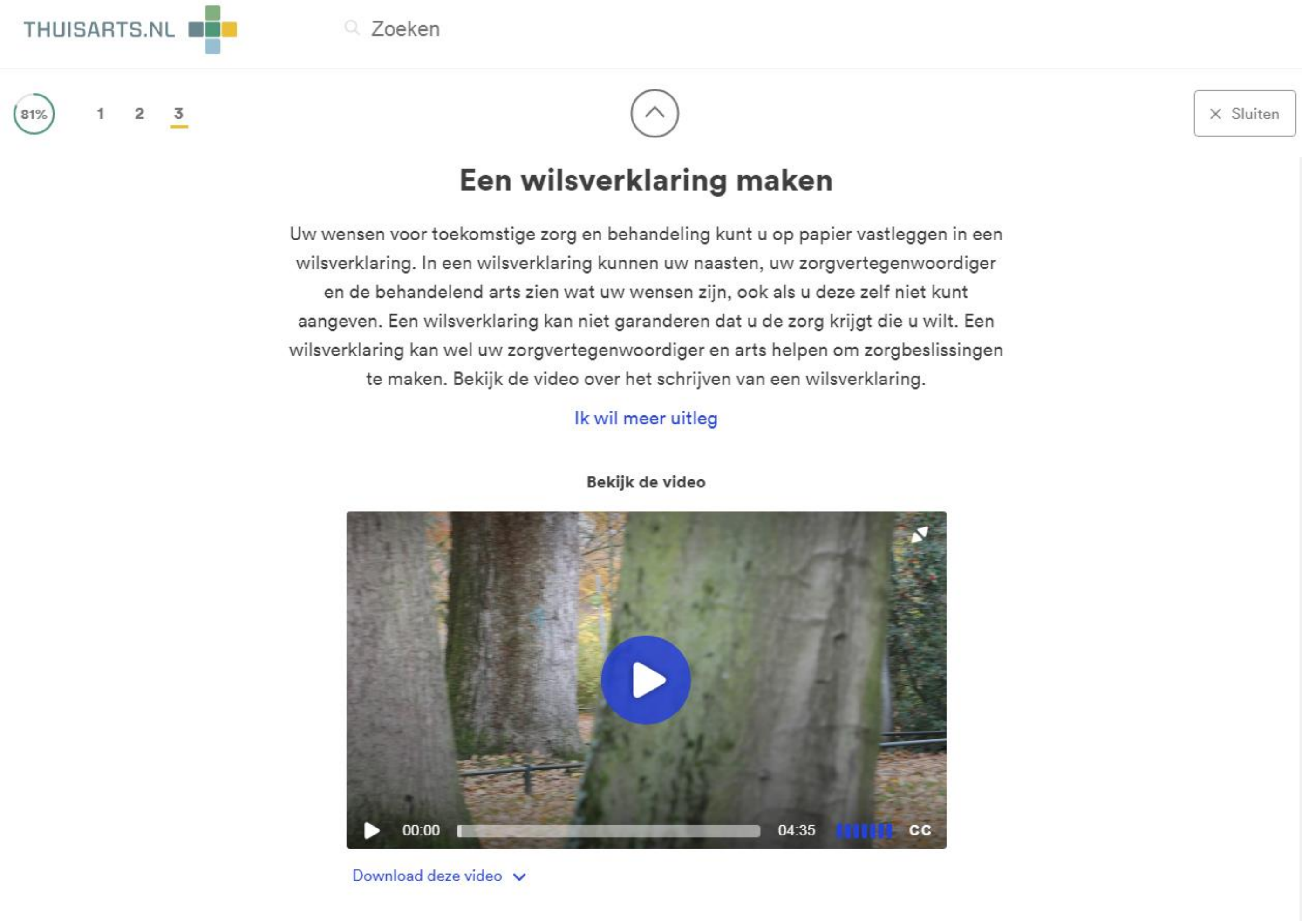

THUISARTS.NL

Zoeken

81% 1 2 3

Sluiten

Een wilsverklaring maken

Uw wensen voor toekomstige zorg en behandeling kunt u op papier vastleggen in een wilsverklaring. In een wilsverklaring kunnen uw naasten, uw zorgvertegenwoordiger en de behandelend arts zien wat uw wensen zijn, ook als u deze zelf niet kunt aangeven. Een wilsverklaring kan niet garanderen dat u de zorg krijgt die u wilt. Een wilsverklaring kan wel uw zorgvertegenwoordiger en arts helpen om zorgbeslissingen te maken. Bekijk de video over het schrijven van een wilsverklaring.

Ik wil meer uitleg

Bekijk de video

00:00 04:35 CC

Download deze video

Step 3: Recording your treatment and care preferences.

Question: When would you review your advance directive?

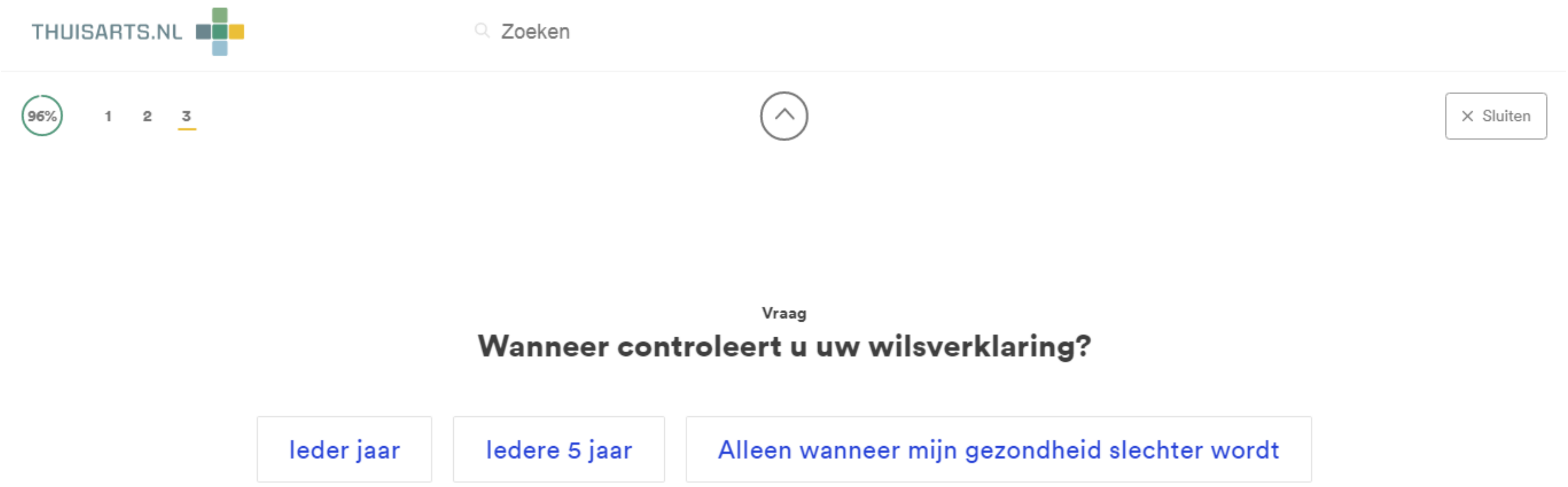

End of program

Your answers as given while completing this program (with the possibility to print the answers or save in PDF).

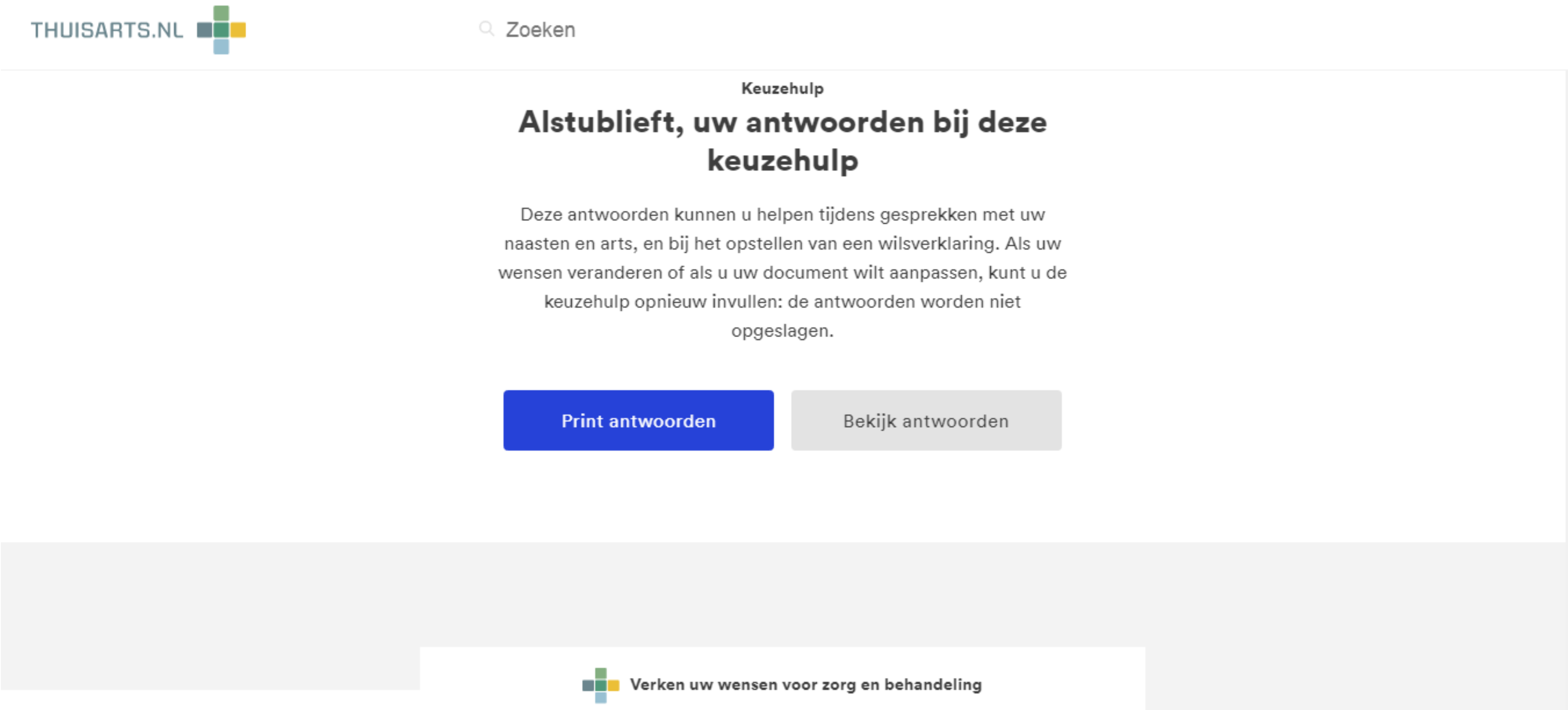

Supplement: Multimedia Appendix 1 [file jmir_v24i12e38561_app1.pdf]
